# Principles and Practice of Surgery

**Published:** 1848-09

**Authors:** 


ART. III.—*Principles and Practice of Surgery.* By the late GEORGE M'CLELLAN, M. D. Edited by his son JOHN H. B. M'CLELLAN, M. D. Grigg, Elliot & Co., No. 14, North Fourth St. 1848. 8vo.

Our readers will remember that it is but little more than one year since we were surprised by intelligence of the sudden death of Dr. Geo. M'Clellan, at the city of Philadelphia. Dr. M'Clellan had been for many years one of the most interesting and successful teachers in that "city of medical schools," and had enjoyed both in his public and private practice extraordinary means of acquiring a practical knowledge of surgery. He was known to be a strong and original thinker, and to the thousands who have listened to his instructions, it is no extravagant laudation, to say that his lectures were always enriched with new and practical suggestions, such that the listener never failed to feel himself abundantly repaid for his time and attention.

It was therefore with the more sincere regret that we heard of his decease, because the world had not only lost one of its most useful men, but that too, as we believed, before he had been able to record his valuable experience. But in this book (of the existence of which we were not then aware) his friends and admirers may find a consolation, yet not unmixed with sorrow; for although it is by no means a complete work upon surgery, such as he was capable of constructing, and such as he had evidently designed, still it is a most precious fragment, and which, like a broken shaft, will serve hereafter to remind all who may look upon it how abruptly his great life was closed.

The work is presented to the public by his son, John H. B. M'Clellan, M. D., and we shall give our readers the explanation of the peculiar circumstances under which it appears, in the editor's own language.

Urged and solicited from year to year by many friends and pupils, to give the results of a valuable experience, it was not until a few months before his death that the author was enabled to commence his long promised undertaking. The constant interference of a large practice, prevented him from writing except at uncertain and irregular intervals. Many of the accompanying pages were penned whilst suffering acutely from disease, and relief from pain was often sought by occupying his mind in recording the views contained in the present volume. The work, therefore, necessarily bears the marks of haste and deficiency of arrangement, mentioned by the author in his preface. As if forewarned by some undefinable impulse, that he might not be spared to carry out his entire object, he seemed bent upon hastily finishing what was meant to be the first volume, and its earlier pages were actually passing through the hands of the printer, whilst he was endeavoring to complete it. Suddenly cut off in the midst of these labors, the whole manuscript was left in much confusion, and many of the subjects in its latter part were incomplete. With little clue to his intentions, other than the knowledge of his opinions, derived fiom some years of daily practice and observation with him, I have endeavored to complete all that was left unfinished, in strict accordance with his peculiar views. Preferring to leave everything as nearly as possible as it came from his hands, I have not felt myself justified in interfering with the distribution or division of the different subjects.

Owing to these circumstances, and to the confusion attending the author's sudden decease, a large portion cf the work has been hurried through the press without receiving the necessary revision, which will account for the large errata that I have appended. To those notes alone which might be thought not altogether connected with the text, I have attached marks to distinguish them as having been inserted by myself. These chiefly refer to important operations performed by the author, which it seemed due to his professional memory, should be mentioned, and which with many others, I trust at some future period to give more at length. I am not without hope, that with these, and a vivid recollection of his opinions, I shall be enabled to carry out the work to the extent originally contemplated. As now published, the chapters on the different subjects treated of are complete in themselves, and contain most of the important principles of Surgery."

To this we shall add, without any apology for so unusual a mode of review, the "Author's Preface," as found among his manuscript papers. It is so eminently characteristic of the man, that its authorship, could never be questioned; and it would be as unpardonable to omit this, as to leave out the portrait from the first page of a biography.

I have always believed and asserted that no inexperienced man ought to be suffered to write a book on Medicine or Surgery. The world is already too full of useless publications, which have been, for almost an age, successively copied from each other. As Horace Walpole, after Lord Bacon himself, is reported to have said in his time, we can now almost wish that another Caliph Omar would arise, and burn up the whole library of compilations and plagiarisms which have been accumulating ever since the grand and blessed fire of bibliopolitan Alexandria. But my present situation before the public is somewhat of an exception to my own rule. I have now arrived at that period of life which I always thought entitled an educated and observing man to be heard; and my old friends and former pupils will give me no farther indulgence in the way of resisting these demands. They all claim of me the published reports of my extemporaneous lectures, delivered years ago during successive winters, in the Jefferson and Pennsylvania Colleges of this city. Some of my most attentive hearers have drawn up abstracts for me, and kindly threaten to print a volume of such matter if I do not gratify them by filling up the manuscript blanks with my own corrections and additions, as I can best recollect how they were originally delivered. When I first promised to undertake this publication, I must confess I thought it would be far easier to complete than it has since proved to be. In looking over the manuscript volumes of my best pupils I find they all mistook many important points and connections of ideas, and left too many gaps and interspaces among the different subjects to enable me to use them with any advantage. I have been obliged, therefore, to shut myself up occasionally from the world, at least for an hour or two at a time, and reproduce my former train of ideas and facts, as I was in the habit of giving them extemporaneously in the lecture room. The constant interruptions, however, which a pressing and busy practice has invariably inflicted upon me, have prevented me trom any methodical distribution of the subjects or discussions. One idea has necessarily grown upon the top of another, until I have in this manner rapidly accumulated the outlines of my views and experience on the principles and practice of Surgery. Perhaps my oldest pupils will discover from the introduction of the cell doctrine and some other late novelties, that I have not altogether neglected the progress of science since the period of their studentship. I am persuaded that they will find I have substantially preserved the spirit and character of my old lectures—to which they used to listen with more patience and satisfaction than I ever deserved to be gratified with. Believing that they will all feel disposed to pardon whatever appearances of haste and carelessness they may detect in these pages, I therefore give my manuscript, written literally *currente calamo*, to the printer, without even a revision.

The volume opens with the *Immediate Effects of Injuries upon the System*, or what is usually termed the "shock," as a circumstance antecedent to inflammation, and when the shock terminates fatally, constituting the sole morbid phenomenon, a subject which the author rightly thinks has been too generally overlooked. It is the first impression of the injury upon the nervous system, and is manifested in a diversity of modes. In some cases the circulating system is chiefly made to suffer, the respiratory in another, or the cerebral, or the digestive, or urinary in others. In all of which there is more or less complete supension of their respective functions, and when most complete the shock is "overwhelming." There is also the "insidious shock," in which although the injury is extensive and severe, yet the patient makes no complaints, the pulse and respiration remain undisturbed and inexperienced medical attendants take no alarm. "Such patients may be compared to the condition of a ship whose rudder has been lost, or whose cable has parted, and a treacherous current is wafting her on rapidly but quietly to the engulphing malstrom." As Hunter remarks, "nature requires to feel the injury," and if in accidents of so grave a character she does not, she will make no effort to repair the injury, and death is almost inevitable.

In accidents less grave, the shock is followed by *reaction*. which constitutes therefore a new subject, and is considered in a seperate chapter:

"In our management of cases of reaction, it is obvious that care should be taken not to allow the proceedure to run too high, or continue for too long a period. Direct depletion, however, can rarely be necessary, except in very full habits, and during very violent excitement. There is always some risk of sudden depletion being followed by a repetition of the prostration, or with what we shall subsequently have occasion to notice under the head of irritative excitement dependent on loss of power to sustain itself. In general, sponging with cool water, fresh air, and a moderate elevation of the head, will sufficiently subdue the excitement following reaction of every kind."

Let us be careful, indeed, how we mistake mere excitement for pure inflammatory reaction, less by the injudicious use of the lancet we increase rather than diminish the excitability, and even produce a fatal exhaustion.

"There can be no greater mistake than to resort to severe depletion, under the idea of preventing inflammation. Such a course will render the system irritable, as well as feeble, and be sure to increase the evil of the very circumstance which it is intended to prevent. The slightest punctures and scratches in habits debilitated by repeated loss of blood and starvation, as in the irritable sympathizing temperament of Hunter, have often produced the most malignant and destructive inflammatory processes. In habitual drinkers, it is almost always necessary to resort to direct and constant stimulation after such evacuations as have already been suggested."

*Irritation* also occurs before the access of inflammation even in such structures as tendons and cartilages, where nothing but vital actions are performed. No definition of irritation is given by our author, beyond the sufficiently vague statement that it is generally believed to be "a mere derangement of the principle of innervation, totally unaccompahied by vascular excitement." "Deranged" action, "changed" action, "perverted" action, a "new" action, are always convenient phrases under which to conceal our ignorance of the character of the change which has occurred, and may under some circumstances be correctly employed; yet it must be confessed that such pathological explanations are exceedingly unsatisfactory as a basis for correct therapeutics.

Some of Dr. McClellans remarks under the head of "Irritation in the brain," remind us that he was a disciple to the doctrines of Phrenology' and that in 1838 he published a case of "Spina Ventosa of the skull,' with the accompanying morbid physiological phenomena and the subsequent surgical operation. In looking at which report the professional reader might be impressed with the exceedingly bad taste shown in the selection of a daily paper, as the vehicle for the communication, the length and egotism of the article, and the complete failure of the Doctor to make of it a "case" for Phrenology. But if he did not by its publication furnish any new evidence in favor of the science of Gall, he certainly did succeed, with the aid of the sore looking wood cut which embellished the report, and of the extensive circulation of the sheet, in informing the public that he practised surgery and sometimes made capital operations in the city of Philadelphia.

The remarks to which I refer, are the following:

"But from the points just alluded to in regard to the diversity of organs in the brain, it must be inferred that a great variety of symptoms prevails in different cases. Some patients will always crave to go home immediately after a shock, no matter where they may be located, and an incessant expression of this desire will continue for hours. Others will contract a sudden aversion for kindred or friends who are present, and will retreat from them with horror, or quarrel without any provocation. Some will pray and beg for divine aid, others will despond and complain of everything that occurs, while a few rare cases will be full of merriment and laughter."

All of which is undoubtedly true, yet it fails utterly to bring any conviction to our mind of the correctness of the doctrine of Gall, that the mind consists of a plurality of independent faculties, each having a seperate and distinct organ for its manifestation. But we shall not stop here to discuss the science of metaphysics, or to arouse phrenology from the deep and eternal slumber into which it is rapidly and inevitably passing. Yet we must be allowed to express our sincere regret that a theory, which, if true would be of such invaluable service to the surgeon, in enabling him with all the precision of geometrical lines to determine to what point of the skull he shall address his remedies, and where apply the trephine has not borne the test of investigation, and become an establiahed truth.

*Irritation of the Urinary Organs* is indicated sometimes, according to our author, by *suppression* of urine. Whether simple *irritation*, without inflammation, is sufficient to cause complete suppression of the urinary secretion or not, it is at least sufficiently certain that the suppression often results from *paralysis*, and indeed we believe this is the most common cause after serious accidents. It is also generally paralysis rather than irritation which causes a retention. And this explanation of the common pathology of suppression accounts for its constituting in most cases so fatal a sign. Dr. McLellan, however, has seen the secretion restored after six days of complete suspension. His remedies are, full doses of spirits of nitre, or Hoffman's anodyne, with turpentine and infusions of diuretic herbs, also calomel, nitre and digitalis in combination, or calomel and camphor. Fomentations and counter-irritants are applied to the back and hips.

*Irritation of the Stomach* forms the subject of the next chapter, in which the treatment must follow the varying conditions of the stomach. *Irritation of the Liver* is manifested by several well known signs; among which the total disappearance of the bile in the secretions, is the most alarming. The principal remedies are cupping, leaching, and counter-irritants, followed by mercurial purgatives and occasionally opiates.

*Irritation of the Heart,* characterized by irregular and febrile contractions of this organ, and also by disturbance of the functions of respiration. In these cases much discrimination is necessary to determine whether the lancet or stimulants are indicated. But the danger is, undoubtedly, that the young practitioner will oftener resort to depletants, than the injudicious use of stimulants. The indications are here well drawn, and are worthy an attentive perusal.

*Irritation in the Nerves,* involves the author in a speculative discussion of little practical utility, in reference to the reciprocal connection between irritation of the nerves of motion and those of sensation, as exemplified in the parallel phenomena of spasms and pain. It seems to be designed as a kind of disquisition upon the subject of irritation and would have been more appropriately arranged before the several chapters on the irritation of special organs. (*To be Continued.*) F. H. H.

